# Erratum

**DOI:** 10.1590/0037-8682-0029B-2024

**Published:** 2025-03-14

**Revista da Sociedade Brasileira de Medicina Tropical/Journal of the Brazilian Society of Tropical Medicine**

Title: Detection of rabies virus in *Callithrix penicillata* (Geoffroy, 1812) in Montes Claros, Minas Gerais State, Brazil 

Vol.:57 | (e00806-2024) | 2024 | doi: https://doi.org/10.1590/0037-8682-0029-2024

**Page 1/1 (e-located)**

e00806-2024

**Should read:**

e00811-2024

